# Atmin mediates kidney morphogenesis by modulating Wnt signaling

**DOI:** 10.1093/hmg/ddu246

**Published:** 2014-05-22

**Authors:** Paraskevi Goggolidou, Nazreen F. Hadjirin, Aggie Bak, Eugenia Papakrivopoulou, Helen Hilton, Dominic P. Norris, Charlotte H. Dean

**Affiliations:** 1 Leukocyte Biology, National Heart and Lung Institute, Imperial College London, London SW7 2AZ, UK; 2 Department of Veterinary Medicine, University of Cambridge, Madingley Road, Cambridge CB3 0ES, UK; 3 College of Nursing, Midwifery & Healthcare, University of West London, Middlesex TW8 9GB, UK; 4 Nephro-Urology Unit, UCL Institute of Child Health, 30 Guilford Street, London WC1N 1EH, UK; 5 Mammalian Genetics Unit, Medical Research Council, Harwell, UK

## Abstract

The DNA damage protein and transcription factor Atmin (Asciz) is required for both lung tubulogenesis and ciliogenesis. Like the lungs, kidneys contain a tubular network that is critical for their function and in addition, renal ciliary dysfunction has been implicated in the pathogenesis of cystic kidney disease. Using the *Atmin* mouse mutant *Gasping6 (Gpg6*), we investigated kidney development and found it severely disrupted with reduced branching morphogenesis, resulting in fewer epithelial structures being formed. Unexpectedly, transcriptional levels of key cilia associated genes were not altered in Atmin*^Gpg6/Gpg6^* kidneys. Instead, *Gpg6* homozygous kidneys exhibited altered cytoskeletal organization and modulation of Wnt signaling pathway molecules, including β-catenin and non-canonical Wnt/planar cell polarity (PCP) pathway factors, such as *Daam2* and Vangl2. Wnt signaling is important for kidney development and perturbation of Wnt signaling pathways can result in cystic, and other, renal abnormalities. In common with other PCP pathway mutants, *Atmin^Gpg6/Gpg6^* mice displayed a shortened rostral-caudal axis and mis-oriented cell division. Moreover, intercrosses between *Atmin^Gpg6/+^* and *Vangl2^Lp/+^* mice revealed a genetic interaction between *Atmin* and *Vangl2*. Thus we show for the first time that *Atmin* is critical for normal kidney development and we present evidence that mechanistically, *Atmin* modifies Wnt signaling pathways, specifically placing it as a novel effector molecule in the non-canonical Wnt/PCP pathway. The identification of a novel modulator of Wnt signaling has important implications for understanding the pathobiology of renal disease.

## INTRODUCTION

Tissue morphogenesis is a critical component of all stages of kidney development and disruption of any of these steps can lead to a variety of developmental defects that impact on kidney function (1,2). Metanephric kidney development initiates at E10.5 in mice when the ureteric bud evaginates from the Wolffian duct and invades the metanephric mesenchyme. Reciprocal signaling between mesenchymal and epithelial cells promotes branching morphogenesis of the ureteric bud, eventually leading to the establishment of nephrogenic progenitors and their subsequent differentiation into mature nephrons containing glomeruli (3,4).

The planar cell polarity (PCP) signaling pathway has emerged as a major and evolutionarily conserved regulator of morphogenetic processes including gastrulation, neurulation and lung branching morphogenesis (5–8). A key function of the PCP pathway is modification of the actin-myosin cytoskeleton to enable morphogenetic movement of tissue, shaping of cells and/or directed cell migration, all of which are critical for normal development and optimum organ function (9–13). In the kidney, perturbation of directed cell movements during tubule morphogenesis, podocyte development and the orientation of cell division and cell adhesion have all been related to defective PCP (14,15). Mutations in the PCP-associated genes Wnt9b and Fat4 have been shown to lead to cyst formation in postnatal kidneys (16,17) while other murine PCP gene mutations that are homozygous lethal, like Vangl2, show early hallmarks of cyst formation such as dilated tubules (9).

The PCP pathway is one of several that can be activated by Wnt ligands. These Wnt-associated pathways have been broadly grouped into the canonical pathway, which is mediated via β-catenin, and two β-catenin independent pathways; the PCP pathway and the Calcium pathway (18). Research has focused on these pathways because both hyper- and hypo-activation of Wnt signaling pathways has been linked to various genetic defects and adult diseases, including renal hypodysplasia, Alzheimer's disease, osteoporosis and cystic kidney disease (18–21). Although the pathways diverge downstream, more proximally, a number of components, including Wnts, Frizzleds and Dishevelleds, are common to more than one pathway (22). While some Wnts appear to predominantly signal via one downstream pathway, e.g. Wnt11 signaling via the PCP pathway, other Wnts important in kidney development, such as Wnt4 and Wnt9b, have been shown to operate through more than one downstream pathway, signaling via both canonical and non-canonical branches (23,24). Ultimately, the intracellular response to a Wnt ligand is determined by precise control of the timing and expression of particular combinations of Wnt ligands, their receptors and the dynamics of these associations.

ATMIN is an essential Zn^+2^ finger protein and was initially identified as a DNA damage response protein (25) involved in the base excision repair pathway and the *in vivo* oxidative stress response (26). *Atmin* has also been found to regulate ciliogenesis in the node by acting as a transcription factor to initiate *Dynll1* gene expression (27). Similarly, an interaction between ATMIN and DYNLL1 has also been observed in several other contexts (28,29). Interestingly, Jurado and colleagues recently established that *Atmin* is a transcriptional regulator of embryonic lung development; acting via its SQ/TQ cluster domain, independently of its role in the DNA damage response. However, the molecular mechanisms by which *Atmin* modulates lung development and its transcriptional targets are not yet known (30). Because of the many links between cilia and cystic kidney disease, as well as the previously established role in lung organogenesis, we wished to investigate whether ATMIN was required for kidney development.

Here we show that ATMIN is critical for normal kidney organogenesis and we reveal a novel role for *Atmin* in modulating Wnt signaling pathways and more specifically, as an effector of the Wnt/PCP pathway.

## RESULTS

### *Atmin^gpg6/Gpg6^* embryos display abnormal kidney morphology

**Figure 1.**
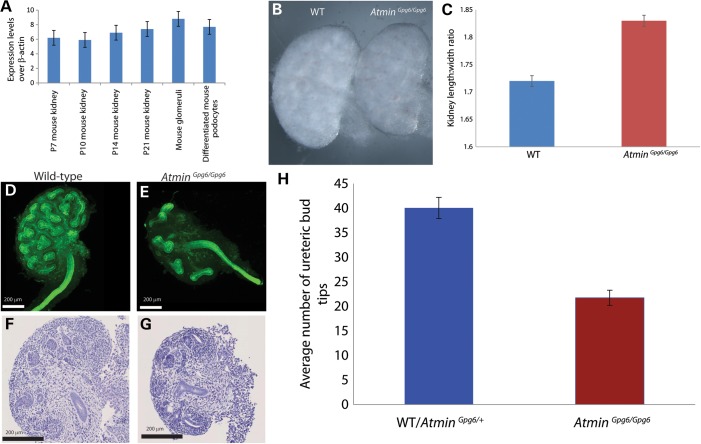
*Atmin* is expressed in the kidneys and its disruption results in kidney morphogenesis defects. (**A**) *Atmin* is expressed in whole mouse kidneys at postnatal day P7, P10, P14 and P21, in isolated mouse glomeruli and in differentiated mouse podocytes. (**B**) *Atmin^Gpg6/Gpg6^* E13.5 kidneys are smaller than WT. (**C**) An increased length to width ratio was observed in *Atmin^Gpg6/Gpg6^* kidneys compared with WT (*n* = 6 per genotype, *P* < 0.01). Wholemount cytokeratin immunostaining of kidneys (**D** and **E**) revealed a reduced number of ureteric bud tips in *Atmin^Gpg6/Gpg6^* (E) compared with WT (D) or heterozygous littermates. Images shown are maximum intensity projections following *z*-stacks through whole kidneys. PAS staining of E13.5 kidneys (**F** and **G**) highlighted histological abnormalities in *Atmin^Gpg6/Gpg6^* (G) compared with WT animals (F). (**H**) Quantification showed an average of 50% fewer bud tips in *Atmin^Gpg6/Gpg6^* kidneys (*n* = 12) compared with WT or heterozygous littermates (*n* = 18; *P* < 0.0001).

Studies have shown that *Atmin^Gpg6/Gpg6^* embryos die around E14 and display a phenotype indicative of a complex ciliopathy including gross edema, exencephaly and left-right patterning defects (31). These mice carry a point mutation (T to A) in the third zinc finger domain of *Atmin*, which causes a serine to cysteine amino acid substitution and results in loss of function (27,31). As a first step in elucidating the potential role of *Atmin* in kidney development, quantitative real-time PCR (qRT–PCR) analysis of *Atmin* expression in kidneys was conducted. *Atmin* expression was detected in cDNA obtained from whole mouse kidney at postnatal days 7, 10, 14 and 21 (Fig. 1A). *Atmin* was also detected in cDNA from mouse glomerular isolates and in differentiated mouse podocytes (glomerular epithelial cells) (Fig. 1A). In addition, *Atmin^Gpg6/Gpg6^* kidneys were examined at E13.5, an early stage of metanephric development, to determine whether kidney development was affected by the *Atmin* mutation in Gpg6. *Atmin^Gpg6/Gpg6^* kidneys were consistently smaller than wild-type (WT) and morphologically distinct (Fig. 1B), displaying an increased length to width ratio (Fig. 1C). Wholemount immunostaining with pan-cytokeratin, to highlight the ureteric bud tree, revealed that branching morphogenesis was severely disrupted in E13.5 *Atmin^Gpg6/Gpg6^* kidneys (Fig. 1E compared with D). Histological examination of E13.5 WT and *Atmin^Gpg6/Gpg6^* littermate kidneys stained with periodic acid-Schiff (PAS) also highlighted the early branching morphogenesis defects in *Atmin^Gpg6/Gpg6^* (Fig. 1G) compared with WT (Fig. 1F). Quantification of this defect from wholemount pan-cytokeratin immunostained kidneys revealed a mean of 22 ureteric bud tips in homozygous kidneys compared with a mean of 40 bud tips in heterozygotes or WT kidneys (Fig. 1H).

**Figure 2.**
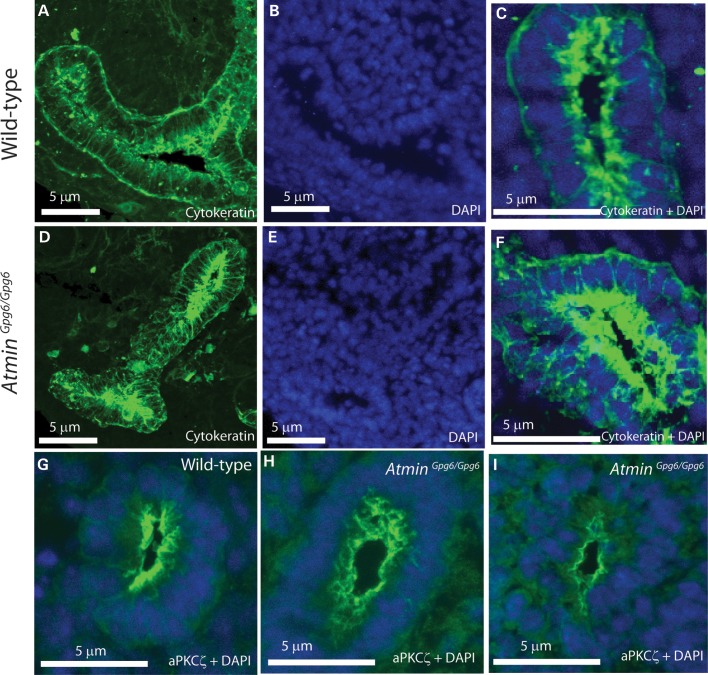
Cytoskeletal differences are observed in *Atmin^Gpg6/Gpg6^* embryos. In *z*-stack images of WT (**A**) and *Atmin^Gpg6/Gpg6^* (**D**) kidney sections immunostained for cytokeratin (green), epithelial cells appear highly disorganized and randomly orientated. (**B** and **E**) Epithelial cells are difficult to distinguish from surrounding mesenchyme by DAPI in *Atmin^Gpg6/Gpg6^* (compare E with B). Cytokeratin stained renal vesicles, with disorganized epithelial cells that are frequently multi-layered and misaligned with respect to one another are observed in the mutant (**F**) compared with WT, where the cells show a regular order and are uniformly aligned with each other (**C**). Normal apical-basal polarity was observed in E13.5 *Atmin^Gpg6/Gpg6^* kidneys (**H** and **I**) and their WT littermates (**G**) as determined by aPKCζ staining of E13.5 whole *z*-stack sections. Images are representative of at least four animals in each category.

More detailed examination of E13.5 kidney sections immunostained with pan-cytokeratin revealed cellular disorganization in *Atmin^Gpg6/Gpg6^*, where the epithelial cells were randomly orientated with respect to each other, rather than the uniform alignment typical of epithelial cells during tubulogenesis. Compared with the well-ordered and neatly arranged epithelial cells in WT sections (Fig. 2A–C), the extent of cellular disorganization in homozygous kidney sections (Fig. 2D–F) meant that epithelial cells were difficult to distinguish from the surrounding mesenchyme by 4′,6-diamidino-2-phenylindole (DAPI) staining alone (compare Fig. 2B with E), without the use of an epithelial cell marker (cytokeratin in Fig. 2A, C, D and F). Since significant disorganization and misalignment of epithelia was observed in *Atmin^Gpg6/Gpg6^*, it was hence important to determine whether apical-basal polarity was disturbed. Despite the overall disruption to epithelial organization in the developing tubules, immunolabelling with the apical membrane marker aPKCζ showed normal apical localization of this protein on the luminal side of tubules in both WT (Fig. 2G) and homozygous kidneys (Fig. 2H and I). The normal localization of aPKCζ indicated there was no overt disruption to apical-basal polarity in *Atmin^Gpg6/Gpg6^* kidneys.

### Cilia architecture and hedgehog signaling are not disturbed in *Atmin^Gpg6/Gpg6^* kidneys

**Figure 3.**
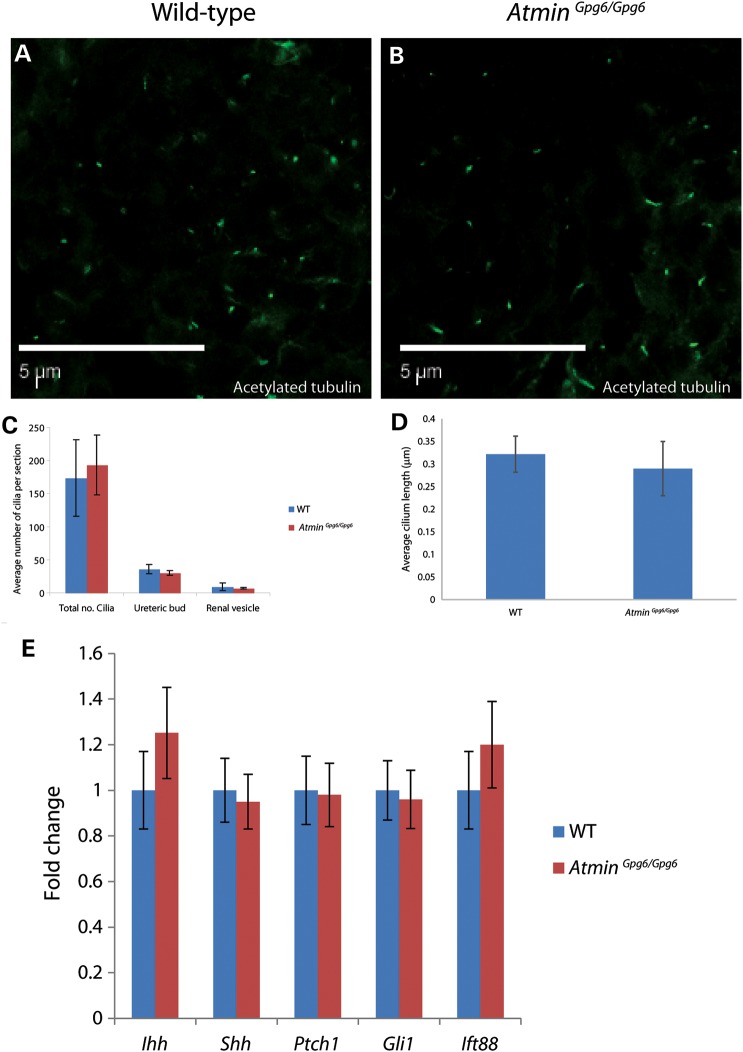
Cilia numbers, cilia length and *Hh* signaling are not affected in *Atmin^Gpg6/Gpg6^* embryos. E13.5 *z*-stack images of WT (**A**) and *Atmin^Gpg6/Gpg6^* (**B**) kidney sections immunostained for acetylated tubulin showed no difference in cilia number or length. (**C**) Quantitation of the number of cilia present at the whole of the E13.5 kidney section or only in the ureteric bud or renal vesicle showed comparable cilia numbers between WT (blue) and Atmin^Gpg6/Gpg6^ (red) E13.5 kidney sections (*n* = 6 per genotype). (**D**) The average cilium length was comparable between WT and *Atmin^Gpg6/Gpg6^* (*n* = 50 per genotype). (**E**) qRT–PCR analysis of E13.5 whole kidneys displayed no significant difference in expression levels for genes relevant to Hh signaling (*Ihh*, *Shh*, *Ptch1*, *Gli1*) and ciliogenesis (*Ift88*) between WT (blue) and *Atmin^Gpg6/Gpg6^* (red; *n* = 6 embryos per genotype).

Recent studies have shown a link between ATMIN and ciliogenesis in the node that leads to perturbation of left-right asymmetry (27). Despite the overall disruption to epithelial organization in the developing kidneys, examination of cilia highlighted by immunofluorescence with acetylated tubulin revealed no obvious difference in cilia morphology between WT and *Atmin^Gpg6/Gpg6^* (Fig. 3A and B). Quantification showed no significant difference in the number of cilia between WT and *Atmin^Gpg6/Gpg6^* whole kidneys, or within ureteric buds or renal vesicles alone (Fig. 3C). Cilia length was also unaltered in *Atmin^Gpg6/Gpg6^* kidneys (Fig. 3D).

Although we did not detect disruption of ciliogenesis, primary cilia are the site for other important cellular functions, including Hedgehog signaling and trafficking of intraflagellar transport molecules. Gene expression studies on E13.5 WT and *Atmin^Gpg6/Gpg6^* kidneys for genes crucial to these functions were also conducted. The levels of *Ihh*, *Shh*, *Ptch1*, *Gli1* and *Ift88* were not significantly different between WT and *Atmin^Gpg6/Gpg6^* kidneys (Fig. 3E). These data show that *Atmin* is likely not required for either ciliogenesis or key cilia-associated functions in the kidney. Nevertheless, consistent with published results *Dynll1* expression was decreased by 80% in *Atmin^Gpg6/Gpg6^* kidneys, whereas *Dynll2* expression remained unchanged (Supplementary Material, Fig. S1).

### *Atmin^gpg6/gpg6^* kidneys exhibit phenotypes consistent with Wnt/PCP pathway disruption

To investigate the underlying cause for the reduced number of epithelial structures in *Atmin^Gpg6/Gpg6^* kidneys, potential alterations in proliferation or apoptosis were investigated. No significant difference was detected in the percentage of proliferating cells in *Atmin^Gpg6/Gpg6^* (Supplementary Material, Fig. S2B, D and I) kidneys compared with WT (Supplementary Material, Fig. S2A, C and I) either in whole kidney sections or when calculating proliferation in epithelial and mesenchymal cells separately (Supplementary Material, Fig. S2J). Similarly, no change in the level of apoptosis was observed in homozygous mutant kidneys (Supplementary Material, Fig. S2F, H and K) compared with WT (Supplementary Material, Fig. S2E, G and K). In addition, investigation of key genes required for kidney branching morphogenesis, *Gdnf* and *Ret,* revealed no change in expression between WT and *Atmin^Gpg6/Gpg6^* kidneys (Supplementary Material, Fig. S2L). The lack of change in cell proliferation, apoptosis or early differentiation markers suggested that the reduced number of epithelial structures and cellular disorganization observed in *Atmin^Gpg6/Gpg6^* kidneys could be due to defective tissue morphogenesis rather than a cell division or apoptosis defect.

**Figure 4.**
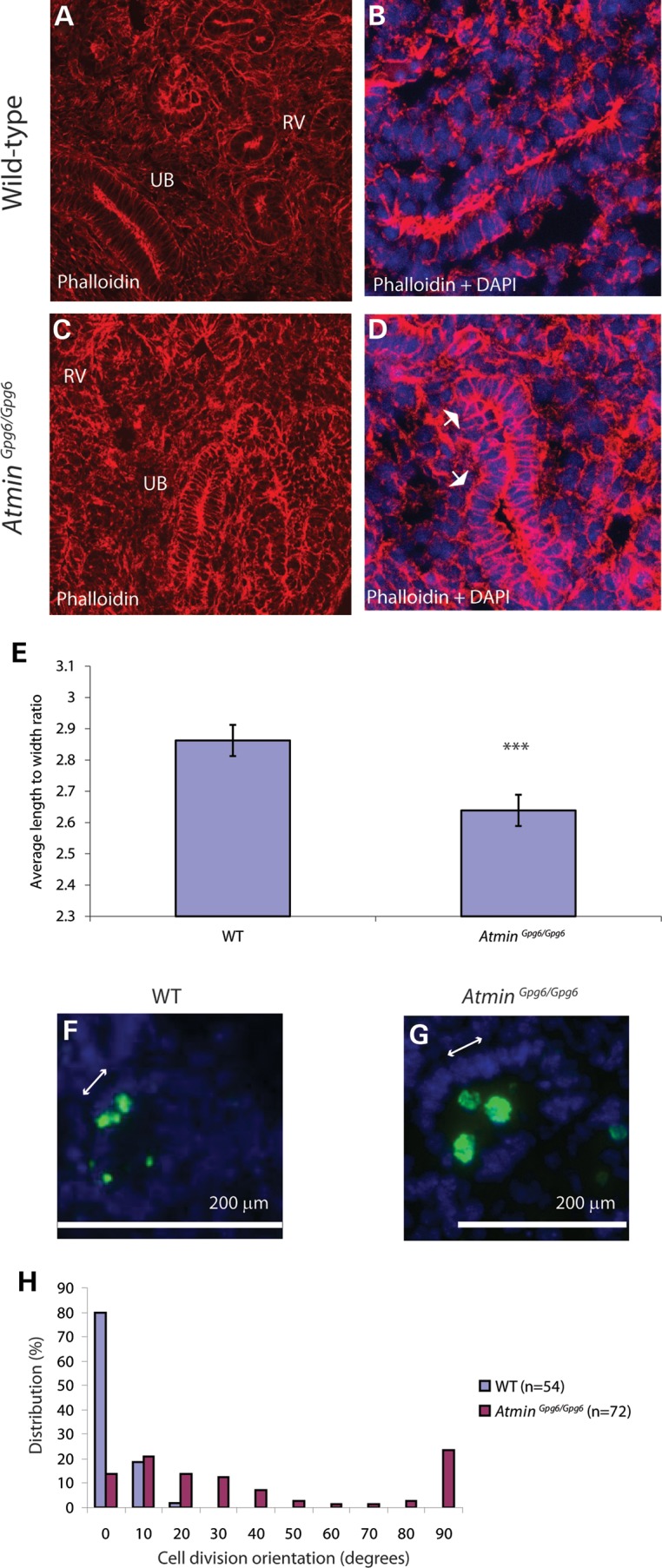
*Atmin^Gpg6/Gpg6^* embryos exhibit cytoskeleton disorganization, decreased length:width ratio and mis-oriented cell division. Phalloidin staining of F-actin (**A**–**D**) reveals altered distribution of filaments in *Atmin^Gpg6/Gpg6^* kidneys (C and D) compared with WT (A and B) RV: renal vesicle, UB: ureteric bud. Expanded, diffuse areas of cortical actin (arrows in D) are visible around cell membranes in *Atmin^Gpg6/Gpg6^* kidneys (D) compared with highly discreet bands of cortical actin in WT littermates (B) (A, C; RV: renal vesicle, UB: ureteric bud). Images are maximum projections of 16 confocal *z* images taken 0.4 µm apart. The average length:width ratio was decreased in *Atmin^Gpg6/Gpg6^* embryos (**E**), compared with WT (WT) littermates (*n* = 8 per genotype, *P* < 0.05 denoted by three stars). Antibodies to phospho-histone H3 were used to mark dividing cells (green in **F** and **G**). In WT embryonic kidneys, cell division (green cells) takes place parallel to the basal membrane that is on the opposite side of nuclei (DAPI, blue) to the lumen (F). In *Atmin^Gpg6/Gpg6^* a more random orientation of cell division is observed (G). Cell divisions were photographed, and the angle between the basal membrane and the orientation of cell division was measured (**H**); angles were grouped into 10° bins. A significantly randomized orientation of cell division is observed in *Atmin^Gpg6/Gpg6^* (*P* < 0.05, Mann–Whitney *U*-test, *n* = 54 for WT, *n* = 72 for *Atmin^Gpg6/Gpg6^*; four embryos per genotype).

Previous studies have shown that the PCP signaling pathway is important for precisely shaping epithelial branches (morphogenesis) during lung and kidney development by affecting cytoskeletal organization (9,10). In addition, the PCP pathway has been linked to regulating cytoskeleton distribution in podocytes (9,11). Moreover, mouse mutants with defective PCP signaling do not show changes in levels of cell proliferation or apoptosis and instead show altered cytoskeleton distribution (10). To investigate whether perturbation of *Atmin* was associated with defective PCP signaling, actin cytoskeletal distribution was compared in E13.5 WT and *Atmin^Gpg6/Gpg6^* kidneys. Phalloidin staining of F-actin revealed diffuse cortical actin in *Atmin^Gpg6/Gpg6^* (Fig. 4C and D), compared with highly discreet cortical actin distribution in WT epithelium (Fig. 4A and B). Markedly expanded areas of F-actin distribution were frequently observed along basal and lateral membranes of *Atmin^Gpg6/Gpg6^* kidney epithelial cells, whereas these areas of substantially expanded actin distribution were never observed in WT kidneys (compare Fig. 4B with D). These changes in actin cytoskeleton distribution are indicative of a disrupted cytoskeleton network. To further investigate whether perturbation of *Atmin* was associated with defective PCP signaling, the average length:width ratio of E13.5 embryos was calculated. A significantly lower length:width ratio was observed in homozygous mutant embryos compared with WT (Fig. 4E; *Atmin^Gpg6/Gpg6^* 2.6, WT 2.8; *n* = 8, *P* < 0.05). A reduced length:width ratio is frequently observed in mouse mutants of PCP-associated genes as a result of convergent extension defects; therefore these results were consistent with *Atmin^Gpg6/Gpg6^* mice manifesting a defective PCP pathway.

### The orientation of cell division is perturbed in *Atmin^Gpg6/Gpg6^* homozygous kidneys

The orientation of cell division is commonly used as a read-out of PCP signaling. It is usual that cell division within kidney tubules takes place parallel to the basal membrane in the *x*-axis, i.e. perpendicular to the tubular longitudinal *y*-axis and it is possible to visualize this either by examining whole tubules or by looking at transverse sections through tubules, where cell division primarily occurs at the luminal surface (32,33). The orientation of epithelial cell division relative to the basal membrane was therefore examined in transverse sections of E13.5 WT and *Atmin^Gpg6/Gpg6^* kidneys. The normal bias toward a uniform orientation of cell division, parallel to the basal membrane, was present in WT kidney sections (Fig. 4F), whereas in *Atmin^Gpg6/Gpg6^*, a much more random cell division orientation was observed (Fig. 4G). Quantification of the angle of division showed 100% of WT cells undergoing cell division at an angle between 0 and 20° from the basal membrane axis (Fig. 4H). In *Atmin^Gpg6/Gpg6^* kidney sections, the angle of cell division was randomly distributed within each of the 10° brackets between 0 and 90° (Fig. 4H).

### *The Atmin^Gpg6^ mutation* affects transcription of key genes in the non-canonical Wnt/PCP pathway

**Figure 5.**
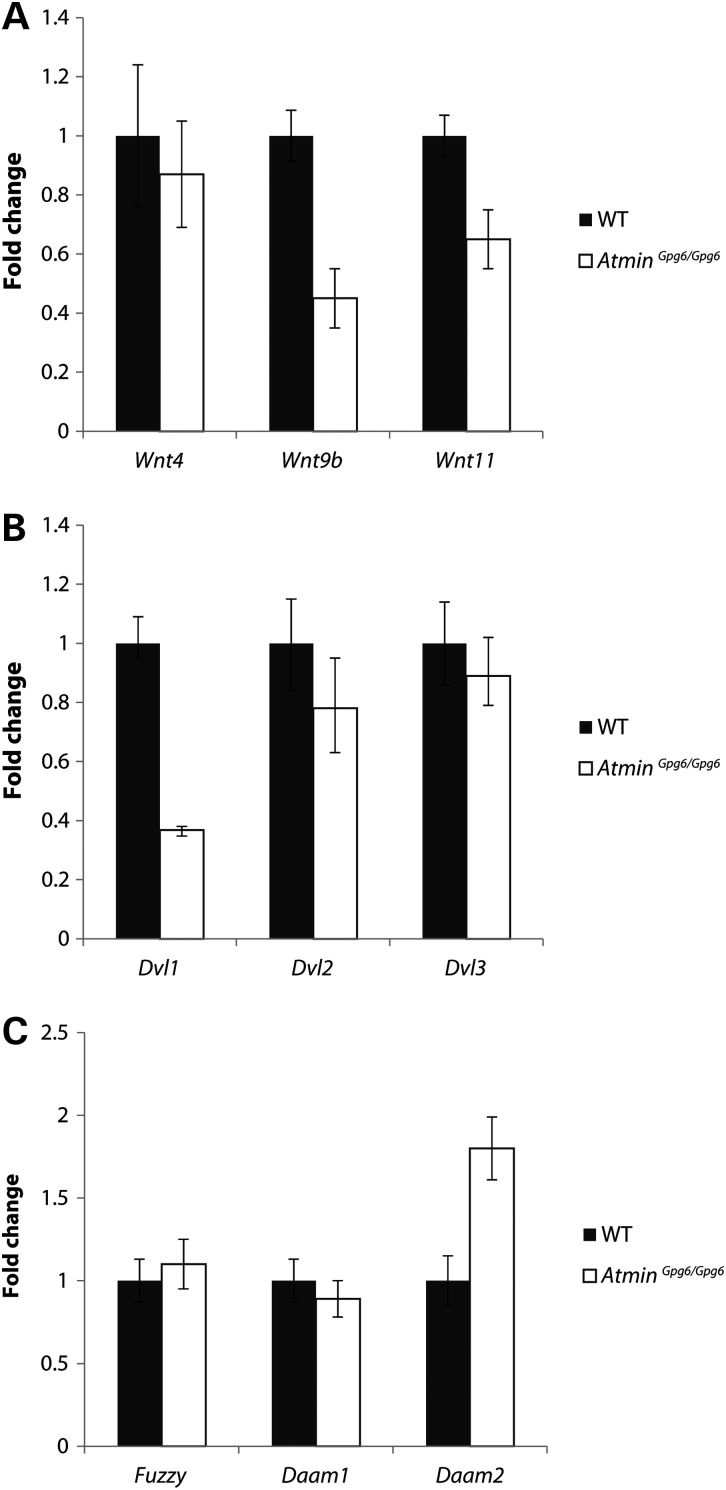
Non-canonical Wnt/PCP gene expression levels are altered in *Atmin^Gpg6/Gpg6^*. Real-time PCR analysis revealed differential gene expression patterns between WT (black) and *Atmin^Gpg6/Gpg6^* (white) E13.5 kidneys. (**A**) Whereas no difference was seen in the expression of canonical Wnt genes (*Wnt4*), *Wnt9b* and *Wnt11*, which are associated with non-canonical/PCP signaling, displayed significantly decreased expression in *Atmin^Gpg6/Gpg6^* compared to WT (*P* < 0.01). (**B**) *Dvl1* expression was decreased by 0.6-fold in *Atmin^Gpg6/Gpg6^* compared with WT littermates (*P* < 0.01), while *Dvl2* and *Dvl3* expression was not significantly altered. (**C**) Expression levels of *Fuzzy* and *Daam1* were not significantly altered, whereas a significant increase in *Daam2* expression was observed in *Atmin^Gpg6/Gpg6^* kidneys compared to WT (*n* = 4, *P* < 0.01).

Because the morphological defects observed in *Atmin^Gpg6/Gpg6^* kidneys were consistent with perturbation of the Wnt/PCP pathway, expression of genes relevant to canonical Wnt and non-canonical Wnt/PCP signaling were examined. No difference in expression was observed for *Wnt4*, a gene most frequently associated with the canonical *Wnt* pathway, whereas the genes *Wnt9b* and *Wnt11* that are usually linked to the non-canonical Wnt/PCP pathway showed decreased expression in *Atmin^Gpg6/Gpg6^* compared with WT (Fig. 5A). Furthermore, a 0.6-fold decrease in the expression of *Dvl1* was observed, whereas the levels of *Dvl2* and *Dvl3* remained unchanged (Fig. 5B). Interestingly, no difference was detected in other PCP pathway genes; *Fuzzy* and *Daam1*, but there was an almost 2-fold increase in *Daam2* expression in *Atmin^Gpg6/Gpg6^*, further emphasizing the involvement of *Atmin* in the PCP/non-canonical *Wnt* pathway (Fig. 5C).

### β-Catenin and Vangl2 proteins are altered in *Atmin^Gpg6/Gpg6^* kidneys

**Figure 6.**
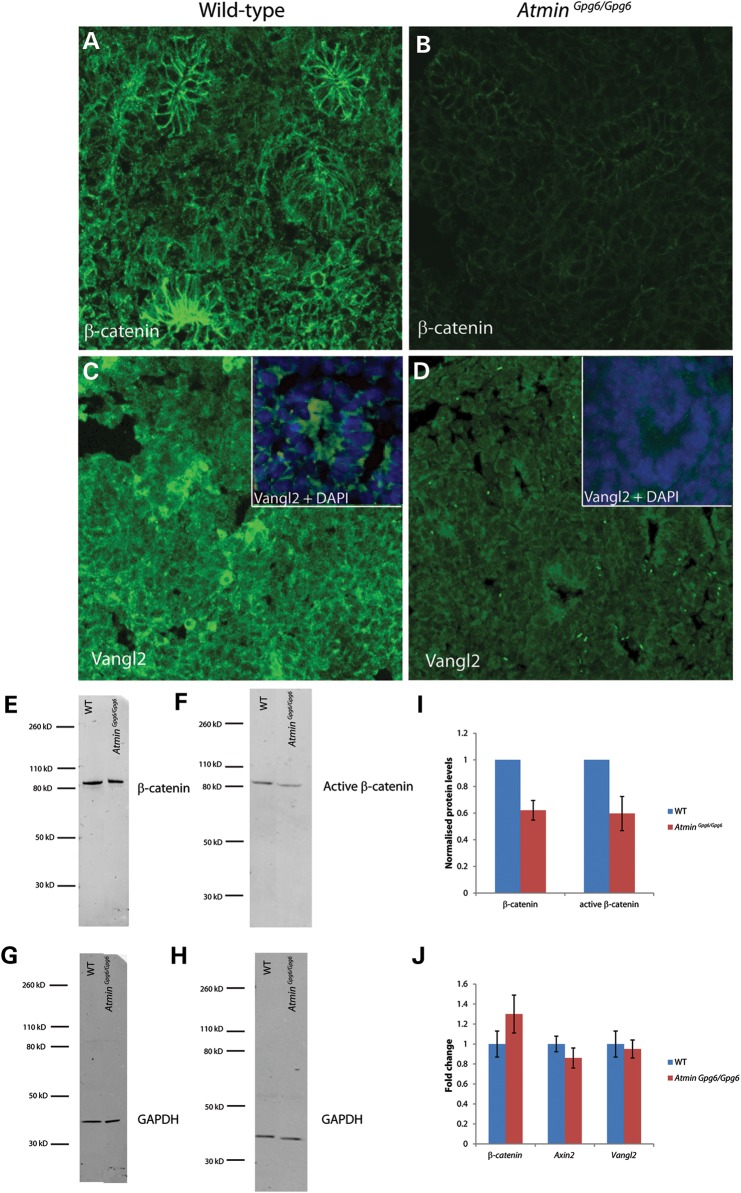
Decreased β-catenin and Vangl2 levels are observed in *Atmin^Gpg6/Gpg6^* E13.5 embryonic kidneys compared with WT littermates. Kidney sections from WT (**A** and **C**) and *Atmin^Gpg6/Gpg6^* (**B** and **D**) embryos were immunostained for β-catenin (A and B) and Vangl2 (C and D). β-Catenin immunostaining was significantly reduced in *Atmin^Gpg6/Gpg6^* (B) compared with WT (A). Vangl2 immunostaining was also dramatically decreased and apical enrichment was altered in *Atmin^Gpg6/Gpg6^* (D) compared with WT (C). Overlays of DAPI and Vangl2 localization emphasize the loss of apical enrichment of Vangl2 protein in *Atmin^Gpg6/Gpg6^* (D inset) versus WT (C inset; *n* = 4). All images are maximum projections of confocal z stacks taken at 0.4 µm intervals. Images were taken in identical conditions and post-acquisition manipulations were identical. Western blotting revealed a 0.4-fold decrease (**I**) in β-catenin (*P* < 0.0001) (**E**) and active β-catenin (*P* = 0.03) (**F**) protein levels compared with WT. GAPDH was used as a loading control (**G** and **H**). *β-catenin, Axin2* and *Vangl2* mRNA expression remained unaltered in E13.5 *Atmin^Gpg6/Gpg6^* embryos compared with WT littermates (**J**, *n* = 4).

Since transcriptional differences relevant to the Wnt signaling pathway were observed between the *Atmin^Gpg6/Gpg6^* and WT embryonic kidneys, the levels of β-catenin and Vangl2, two key proteins for the canonical and non-canonical/PCP *Wnt* signaling pathways, respectively, were also examined. A striking decrease in β-catenin was seen in *Atmin^Gpg6/Gpg6^* by immunofluorescence (Fig. 6B) compared with WT (Fig. 6A) and western blotting confirmed a 40% reduction in both total and active β-catenin (Fig. 6E, F and I). An overall decrease in Vangl2 immunostaining was also observed in *Atmin^Gpg6/Gpg6^* kidney sections (Fig. 6D) compared with WT (Fig. 6C) and significantly, apical enrichment of Vangl2, which is required for its proper function, appeared considerably reduced in *Atmin^Gpg6/Gpg6^* (compare insets in Fig. 6C and D). Intriguingly, similar levels of both β*-catenin* and *Vangl2* transcripts were observed in *Atmin^Gpg6/Gpg6^* and WT kidneys (Fig. 6J), indicating that *Atmin* does not directly act to alter these molecules at the transcriptional level. Similarly, no change was detected in expression levels of *Axin2*, another critical gene in the canonical Wnt pathway, between WT and *Atmin^Gpg6/Gpg6^* kidneys (Fig. 6J). Nevertheless, loss of *Atmin* function specifically affects both β-catenin and Vangl2 proteins.

### *Atmin* genetically interacts with *Vangl2*

**Figure 7.**
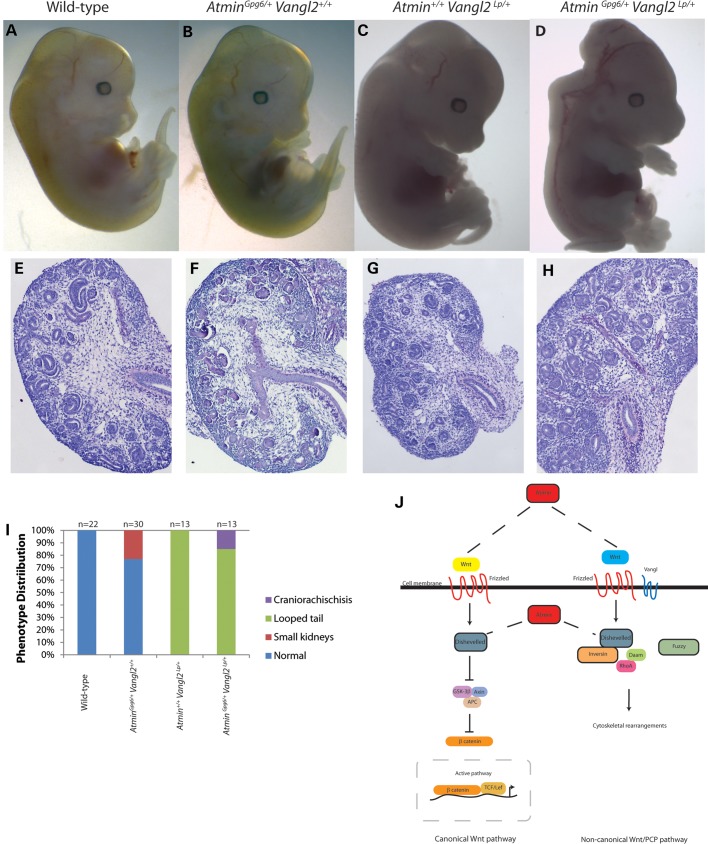
*Atmin^Gpg6^* mutants genetically interact with *Vangl2^Lp^*. Intercrosses of *Atmin^Gpg6/+^* with *Vangl2^Lp/+^* generated double heterozygous embryos, with a percentage of double heterozygotes displaying craniorachischisis (**D**), a phenotype that was never observed in WT (**A**), *Atmin^Gpg6/+^Vangl2^+/+^* (**B**) or *Atmin^+/+^Vangl2^Lp/+^* (**C**) E13.5 embryos. The number of double heterozygotes displaying craniorachischisis compared with other genotypes is statistically significant (*P* < 0.05). *Atmin^Gpg6/+^Vangl2^Lp/+^* E13.5 kidneys (**H**) appeared delayed without clear demarcation between the cortical and medullary regions that was evident in the other three genotypes (**E**–**G**). (**I**) Graphical representation of the percentage distribution of embryonic phenotypes collected from intercrosses. The number of embryos examined per genotype is indicated at the top of each column. A small decrease in kidney size was observed in 20% of *Atmin^Gpg6/+^* mice. (**J**) A schematic diagram representing potential modes of interaction between Atmin and Wnt signaling.

Taken together, our results showed that the phenotype observed in the *Atmin^Gpg6/Gpg6^* kidneys most closely resembled that of other PCP pathway mutants. Furthermore, our data suggested that *Atmin* and *Vangl2* might genetically interact; this possibility was tested by conducting intercrosses between *Atmin^Gpg6/+^* and *Vangl2^Lp/+^* heterozygotes (Fig. 7A–D and I). Fifteen percent of double heterozygotes exhibited craniorachischisis, a similar level to that previously observed between *Vangl2* and another PCP associated factor *Ptk7* (34) (Fig. 7I). In contrast, mild architectural kidney defects were observed even in *Atmin^Gpg6/+^*; *Vangl2^Lp/+^* embryos that genotyped as double heterozygotes but did not show craniorachischisis. Specifically, WT kidneys contained clearly distinguishable cortex and medullary regions (Fig. 7E). *Atmin^Gpg6/+^* (Fig. 7F) and *Vangl2^Lp/+^* (Fig. 7G) single heterozygote kidneys also showed distinct cortex and medullary regions; however, this distinction was not visible in *Atmin^Gpg6/+^*; *Vangl2^Lp/+^* (Fig. 7H). This phenotype is indicative of a differentiation defect and a similar lack of cortico-medullary definition is observed in kidneys homozygous for the *Lp* mutation in *Vangl2* (9). Our data show that *Atmin* and *Vangl2* genetically interact; moreover the disparity in the number of double heterozygous embryos exhibiting craniorachischisis and kidney defects suggests that the Atmin-Vangl2 interaction may be more important in some specific aspects of embryo development, such as kidney formation, than in others, e.g. neural tube closure. The genetic interaction between *Atmin* and *Vangl2* revealed in these experiments provides additional evidence that *Atmin* is important for modulating the Wnt/PCP pathway in normal kidney development.

## DISCUSSION

### *Atmin* and kidney development

The molecular and phenotypic analyses of *Atmin^Gpg6/Gpg6^* embryos presented in this manuscript reveal that ATMIN is required for normal kidney organogenesis, with homozygous mutants displaying defective branching morphogenesis leading to formation of fewer epithelial bud tips. Furthermore, we observe disrupted cytoskeleton organization in *Atmin^Gpg6/Gpg6^* kidneys, as has previously been shown for other PCP pathway mutations (9,12,35). ATMIN is also required for lung organogenesis, another organ that undergoes branching morphogenesis to form the tubular network of airways (30). As in the lungs, factors affecting epithelial tube formation can impact on organ function; for example, in the kidneys, there is a direct relationship between impaired development, resulting in generation of fewer glomeruli, and reduced kidney function (36–38).

### *Atmin* regulates Wnt signaling pathways

Wnts are required for many aspects of kidney development and homeostasis; the PCP pathway branch regulates morphogenesis of metanephric mesenchyme and ureteric bud-derived structures, including podocytes, through cytoskeleton reorganization/rearrangement (9,11,12). Our investigation reveals that *Atmin* plays a role in modifying Wnt signaling pathways; in particular *Atmin^Gpg6/Gpg6^* embryos exhibit defects associated with PCP pathway disruption including a shortened rostral-caudal axis, disrupted epithelial organization, altered cytoskeleton distribution and mis-orientation of cell division.

Although several aspects of *Atmin^Gpg6/Gpg6^* embryos resemble those of other PCP pathway-associated mutants such as Vangl2 and Wnt5a, there are also some distinct differences in the phenotypes. For example, homozygous core PCP pathway mutations like Vangl2 or Celsr1 usually result in the severe neural tube defect, craniorachischisis, whereas the *Atmin* homozygotes show exencephaly, a different neural tube closure defect (30,31,39). Moreover the genetic interaction that we observed between *Atmin* and *Vangl2* is not as frequent as for some other ‘core’ PCP pathway genes, where the protein products have been shown to bind directly to each other. Instead, the percentage of embryos exhibiting a genetic interaction indicates that ATMIN may function as an effector molecule in the kidney, in a similar manner to other PCP effectors important in some, but not all, contexts, e.g. PTK7 (34). A similarly low percentage of Vangl2/PTK7 double heterozygotes exhibit craniorachischisis (34) but nevertheless, there is ample evidence that PTK7 makes an important contribution to the PCP signaling pathway (40,41). Interestingly, our analysis of Wnt pathway genes showed altered gene expression of key Wnts important for kidney development, as well as a specific reduction in *Dvl1* but not in *Dvl2* or *Dvl3*. Wnt signaling pathways are activated by the binding of a Wnt ligand to a membrane bound, usually Frizzled, receptor. Subsequently, recruitment of one of the *Dvl* genes to the Wnt/frizzled complex occurs prior to recruitment of additional proteins more specific to a particular downstream signaling branch; for example the canonical or PCP pathway. Since a dramatic decrease was detected in both β-catenin (canonical Wnt pathway-specific) and Vangl2 (PCP pathway-specific) proteins, this indicates that the transcriptional changes seen in *Atmin* homozygous kidneys impact on more than one downstream pathway. Notably, most of the transcriptional changes that we do see are in upstream components of Wnt signaling pathways, i.e. selected Wnt ligands and *Dvl1*. Downstream of this, we see effects on both canonical and non-canonical pathway components; we therefore propose that ATMIN likely influences Wnt signaling pathways somewhere upstream, at the level of one or more of the common Wnt pathway components (Fig. 7J). However, whether or not Atmin is a direct transcriptional regulator of one or more common Wnt signaling pathway components is yet to be determined.

Although an exhaustive study of the expression and function of all the individual components of canonical and non-canonical Wnt signaling is beyond the scope of the current manuscript, our results have revealed some interesting findings about the effect of the *Atmin^Gpg6^* mutation on components of Wnt signaling pathways. For example, a number of studies have highlighted mutual antagonism between different downstream Wnt signaling pathways within the same tissue/cell-type. In this model, canonical and non-canonical Wnts activate one downstream pathway, while simultaneously repressing the other due to competition for Frizzled binding. This is thought to be achieved by Wnts associating with pathway-specific co-receptors such as Lrp6 and Ror2 (42). Consequently, the finding that both β-catenin and Vangl2 proteins were perturbed in *Atmin^Gpg6/Gpg6^* kidneys was somewhat unexpected; however, much of the work showing mutual antagonism of Wnt signaling pathways has been conducted *in vitro* and it is now becoming clear that at the level of tissues or whole organs, the picture is much more complex. *In vivo*, rather than distinct signaling pathways operating separately, interactions between pathways appear much more intricate with integration occurring at many different levels (43). This complexity is evident when looking at Wnt pathway regulation in human disease; for example, studies of Meckel-Gruber syndrome associated proteins have revealed defects in either canonical or non-canonical Wnt signaling depending on the particular mouse model (44). In chronic obstructive pulmonary disease (COPD) both over- and under-expression of β-catenin has been associated with disease (45,46). Moreover, a recent study looking at global gene expression changes in COPD noted an enrichment of Wnt pathway genes; importantly, the majority of both canonical and non-canonical pathway components were simultaneously downregulated (47). This study further emphasizes the complexities of Wnt signaling in organogenesis.

### *Daam2* is specifically upregulated in *Atmin^gpg6/gpg6^* kidneys

The functions of the *Dishevelled associated activator of morphogenesis (Daam)* genes 1 and 2 are still not well understood but, as inferred by their name, they associate with Dvls and usually operate in the PCP pathway to regulate cytoskeleton dynamics (see schematic in Fig. 7J). Work in *Xenopus* showed that *Daam1* knockdown alters morphogenesis of the pronephric tubules and collecting duct but does not result in an obvious cystic phenotype (7). In chick, Daam2 has been suggested to modulate formation and stabilization of Wnt/receptor complexes (48). Previous mouse studies have identified a critical role for *Daam1* in heart morphogenesis and for *Daam2* in the developing gut (49,50) and *in vitro*, addition of exogenous Wnt5a led to re-distribution of Daam1 concomitant with increased stress fiber formation in both mouse and human podocyte cells (11). Collectively, these investigations highlight the importance of the *Daam* genes in cell and tissue morphogenesis during organ development. Interestingly, the expression of *Daam1* and *2* in most organs is highly restricted and non-overlapping. For example in the lungs, *Daam2* is restricted to the mesenchyme, whereas *Daam1* is epithelial specific (51). According to the Genitourinary Development Molecular Anatomy Project, GUDMAP database, *Daam1* shows little if any expression in kidneys, while *Daam2* is present at much higher levels and is restricted to the stroma and vasculature. The differing patterns and levels of *Daam1* and *2* in the kidneys indicate that here, *Daam2* is the more important family member and it is therefore unsurprising that in *Atmin^gpg6/gpg6^* kidneys a specific change in *Daam2* alone is observed.

### ATMIN, DYNLL1 and cilia interactions

The *Atmin^Gpg6^* mutation was recently associated with ciliogenesis, specifically in the node, where reduced cilia length is thought to be the cause of the left-right patterning defects present in the embryos (27). However, in the same manuscript cilia in the limbs and neural tube revealed mild structural and no functional defects. Similarly, we did not observe any difference in cilia length or number in *Atmin^Gpg6/Gpg6^* kidneys and consistent with this data, levels of the key cilia gene *Ift88* were unaltered in the homozygotes. Despite the absence of changes in cilia structure, the possibility remained that signaling pathways associated with primary cilia might be affected. However our investigation of one such signaling pathway, the Hh pathway, did not show any difference between *Atmin^Gpg6/Gpg6^* and WT kidneys. In fact, investigations have suggested an association between Wnt signaling and primary cilia structure and function, though this remains a controversial area that has not yet been definitively proven (14,21,52–55).

Another key protein interaction that has been shown in several contexts is that between ATMIN and the dynein motor protein, DYNLL1 (27–29). Consistent with previous studies, downregulation of *Dynll1* but not *Dynll2* was detected in *Atmin^Gpg6/Gpg6^* kidneys. DYNLL1 can function in cilia but also as part of the cytoplasmic dynein complex. In the cytoplasm, DYNLL1 is important for transport of cargo, carrying them toward the minus ends of microtubules and can also be found at the cortex, where it pulls on microtubules attached to the spindle poles during mitosis (56). Although DYNLL1 is both axonemal and cytoplasmic, the fact that no defects were observed in the Hedgehog pathway or in cilia size suggests that in the kidneys, the function of ATMIN is not mediated via the cilia. For PCP to occur, associated proteins must be trafficked to the appropriate ‘sides’ of the cells where they form multi-protein complexes to assert their effects. In addition, a key role of the PCP pathway is to ensure the correct positioning of the microtubule organizing center and basal body docking. It is therefore possible that in the kidney, the effect of disrupting ATMIN is to disturb the cytoplasmic functions of DYNLL1 and thereby disrupt planar polarity pathway functions (either protein trafficking and/or orientation of mitosis). However, an alternative possibility is that mutations in *Atmin* may lead to direct disruption of the actin-myosin cytoskeleton, since abnormal cytoskeletal organization was observed in the homozygous kidneys. In support of this idea, another PCP effector molecule, *Wdpcp*, was recently shown to be required for ciliogenesis but interestingly, Cui *et al*. (13) established that the PCP defects in *Wdpcp* mutants are not due to abnormal ciliogenesis; rather they result from direct perturbation of the actin cytoskeleton, as we have observed in *Atmin^Gpg6/Gpg6^ kidneys*.

Taken together, the molecular and phenotypic analysis of *Atmin^Gpg6/Gpg6^* embryos presented in this manuscript reveals that ATMIN is an important regulator of Wnt signaling pathways during kidney development. Given the critical role of Wnt signaling in kidney development and diseases, the identification of a novel modulator has important implications both for further studies of Wnt signaling pathways and for the design of potential therapeutic strategies.

## MATERIALS AND METHODS

### Mice

The ENU-derived *Atmin^Gpg6^* mice were identified in an ENU mutagenesis screen at MRC Harwell, as previously described (31). *Gpg6* mice show a T to A transversion in exon 3 of *Atmin*, correlating with the third Zinc Finger. This results in a cysteine to serine substitution in the fourth canonical residue (27). *Looptail* mice (Jackson Laboratories, Bar Harbour, ME, USA) carry a point mutation at position 464 that results in a serine to asparagine transition (57,58). Congenic lines of *Vangl2^Lp^* and *Atmin^Gpg6^* mice were maintained on a C3H/HeH background. *Looptail* homozygotes were identified by craniorachischisis and heterozygotes by the presence of a looped tail. Genotyping was carried out using pyrosequencing assays to directly amplify the mutations; WT littermates were used as controls (primers available on request).

### Histology and immunostaining

Kidneys were fixed in 4% paraformaldehyde, dehydrated, wax-embedded and sectioned at 5 µm. In some experiments, PAS staining was used to help distinguish epithelial and mesenchymal components. Immunohistochemistry was performed using 10 µm cryosections for the following antibodies to: phospho-histone H3 (pH3; Millipore, Dundee, UK), Cleaved caspase-3, β-catenin (Cell Signalling Technology, Danvers, MA, USA), rhodamine-conjugated phalloidin (Invitrogen, Paisley, UK), acetylated tubulin, pan-cytokeratin (Sigma-Aldrich, UK), aPKCζ (Santa Cruz Biotechnology, Dallas, TX, USA), Vangl2 (raised in rabbit against the following Vangl2 specific peptide: CLAKKVSGFKVYSLGEENST, 21st Century Biochemicals, MA, USA. See Supplementary Material, Fig. S3 for validation). Bound antibodies were detected with either Alexa Fluor 488 or 594 secondary antibodies (Invitrogen). Cell nuclei were visualized by staining with DAPI. As negative controls, primary antibodies were omitted.

### Quantification of kidney cilia numbers and length

Representative, comparative E13.5 *z*-stack images of kidney sections immunostained for acetylated tubulin from six WT and six *Atmin^Gpg6/Gpg6^* embryos were counted for total, ureteric bud and renal vesicle cilia in Adobe Photoshop. The average cilium length was estimated from the same images as described above, for 50 cilia for each genotype using the line segment tool in Adobe Illustrator. The average (mean) number of cilia or the average cilium length for each genotype was calculated in Microsoft Excel.

### Western blotting

Immunoblotting was carried out using 10 µg/lane of E13.5 WT and *Atmin^Gpg6/Gpg6^* whole kidney protein extract (*n* = at least 9 per genotype run on 3 independent blots); using total β-catenin antibody (Sigma), 1:4000; or non-phospho β-catenin antibody (Cell Signaling Technology), 1:1000. GAPDH (Abcam), 1:5000 was used for loading control.

### Proliferation and apoptosis analysis

Proliferation and apoptotic indices were calculated by counting the numbers of phospho histone-H3-, or cleaved caspase 3-expressing cells, respectively, in kidney sections from three individuals of each genotype, as a percentage of total (DAPI-stained) nuclei. Ki67 staining was used for assessment of percentage proliferation in epithelium and mesenchyme compartments separately. Images are representative of at least three animals for each genotype.

### Quantitative RT–PCR

To measure *Atmin* expression levels, 500 ng of RNA was isolated from either whole CD1 mouse kidneys at P7, P10, P14 or P21, isolated glomeruli or differentiated podocytes using TRI Reagent and cDNA prepared for PCR using the iScript cDNA synthesis kit (Biorad) as per manufacturer's instructions. *Atmin* levels are shown relative to the level of the housekeeping gene β-actin and all assays were performed in triplicate. Glomerular isolations were performed using magnetic beads as previously described (38). Differentiated mouse podocyte cells (gift from Peter Mundel, Harvard Medical School, USA) were cultured on tissue culture plastic coated with 1% Matrigel substrate. For on-going proliferation, cells were cultured at 33°C in a 5% CO_2_ incubator in RPMI (Invitrogen) supplemented with 10% FCS (Invitrogen), antibiotics and IFN-γ (10 U/mL). To induce differentiation, cells were thermoshifted to 37°C and cultured in RPMI in the absence of IFN-γ for 14 days.

To compare gene expression levels in WT and *Atmin^Gpg6/Gpg6^*, one µg RNA was isolated from WT and *Atmin^Gpg6/Gpg6^* E13.5 littermate kidneys using the RNeasy mini kit (QIAGEN) and cDNA was prepared for qRT–PCR using the High Capacity cDNA Reverse transcription kit (ABI). Quantitative real-time PCR was performed for the genes *Ihh, Shh, Ptch1, Gli1, Ift88, Dynll1, Dynll2*, *Gdnf*, *Ret*, *Wnt4*, *Wnt9b*, *Wnt11*, *Dvl1*, *Dvl2*, *Dvl3*, *Daam1*, *Daam2*, *Fuzzy*, *Vangl2, Axin2* and β*-catenin*. All assays were provided by ABI. Alterations in gene expression in *Atmin^Gpg6/Gpg6^* were expressed relative to the mean intensity in WT embryos over β-actin expression, which was given a standardized value of 1. Negative controls of reactions without cDNA template were included. All qRT–PCR assays were performed in triplicate on at least four different embryos of each genotype. Primer details are available upon request.

### Statistical methods

Data were analyzed using unpaired two-tailed *t*-tests, unless otherwise stated. Significance was accepted at *P* < 0.05; error bars in all data represent standard error mean.

## SUPPLEMENTARY MATERIAL

Supplementary Material is available at *HMG* online.

## FUNDING

This work was supported by National Heart and Lung Institute, Imperial College start-up funds (C.H.D. and P.G.), a Wellcome Trust fellowship for MB/PhD graduates (JP) and MRC Harwell core funds (C.H.D, D.P.N and P.G.). Funding to pay the Open Access publication charges for this article was provided by Imperial College.

## Supplementary Material

Supplementary Data
