# Supplementary material for: Atmin mediates kidney morphogenesis by modulating Wnt signaling
Source: Hum Mol Genet. 2014 May 22;23(20):5303–16. doi: 10.1093/hmg/ddu246 (PMC4168818; doi:10.1093/hmg/ddu246)
Supplement: Supplementary Data [file supp_23_20_5303__index.html]

Atmin mediates kidney morphogenesis by modulating Wnt signalling — Atmin mediates kidney morphogenesis by modulating Wnt signaling — Atmin mediates kidney morphogenesis by modulating Wnt signaling — Supplementary Data 

# Atmin mediates kidney morphogenesis by modulating Wnt signaling

## Supplementary Data

Supplementary Data

**Files in this Data Supplement:**

- Supplementary Data - Doc file
