# Supplementary material for: Atmin mediates kidney morphogenesis by modulating Wnt signaling
Source: Hum Mol Genet. 2014 May 22;23(20):5303–16. doi: 10.1093/hmg/ddu246 (PMC4168818; doi:10.1093/hmg/ddu246)

Supplemental Fig. 1. A substantial decrease in *Dynll1* expression is observed in E13.5 *Atmin^Gpg6/Gpg6^* kidneys, while *Dynll2* expression remains unchanged.

Consistent with previous observations, *Dynll1* expression is decreased in *Atmin^Gpg6/Gpg6^* (p<0.01, denoted by ***), while *Dynll2* levels are similar between *Atmin^Gpg6/Gpg6^*(red bars) and wild type (blue bars).

Supplemental Fig. 2. Proliferation and apoptosis are unaffected in *Atmin^Gpg6/Gpg6^* embryos.

Analysis of the percentage of proliferating cells in E13.5 whole kidney sections by immunostaining with anti-phospho histone H3 (A, B) or of apoptosis with anti-cleaved caspase 3 (E, F) and DAPI (C, D, G, H) revealed no significant differences in proliferation (I,J) or apoptosis (K) between wild-type (blue) and *Atmin^Gpg6/Gpg6^* (red; n=4 per genotype). No change in *Gdnf* and *Ret* expression was observed between wild-type and *Atmin^Gpg6/Gpg6^* E13.5 kidneys by qRT-PCR (L, n=4 per genotype).

Supplemental Fig. 3. Vangl2 antibody validation by Western Blotting.

A band of 75 kDa, representing the GFP-tagged Vangl2 construct is detected in HEK293 cells. An identical band is detected using an anti-GFP antibody.


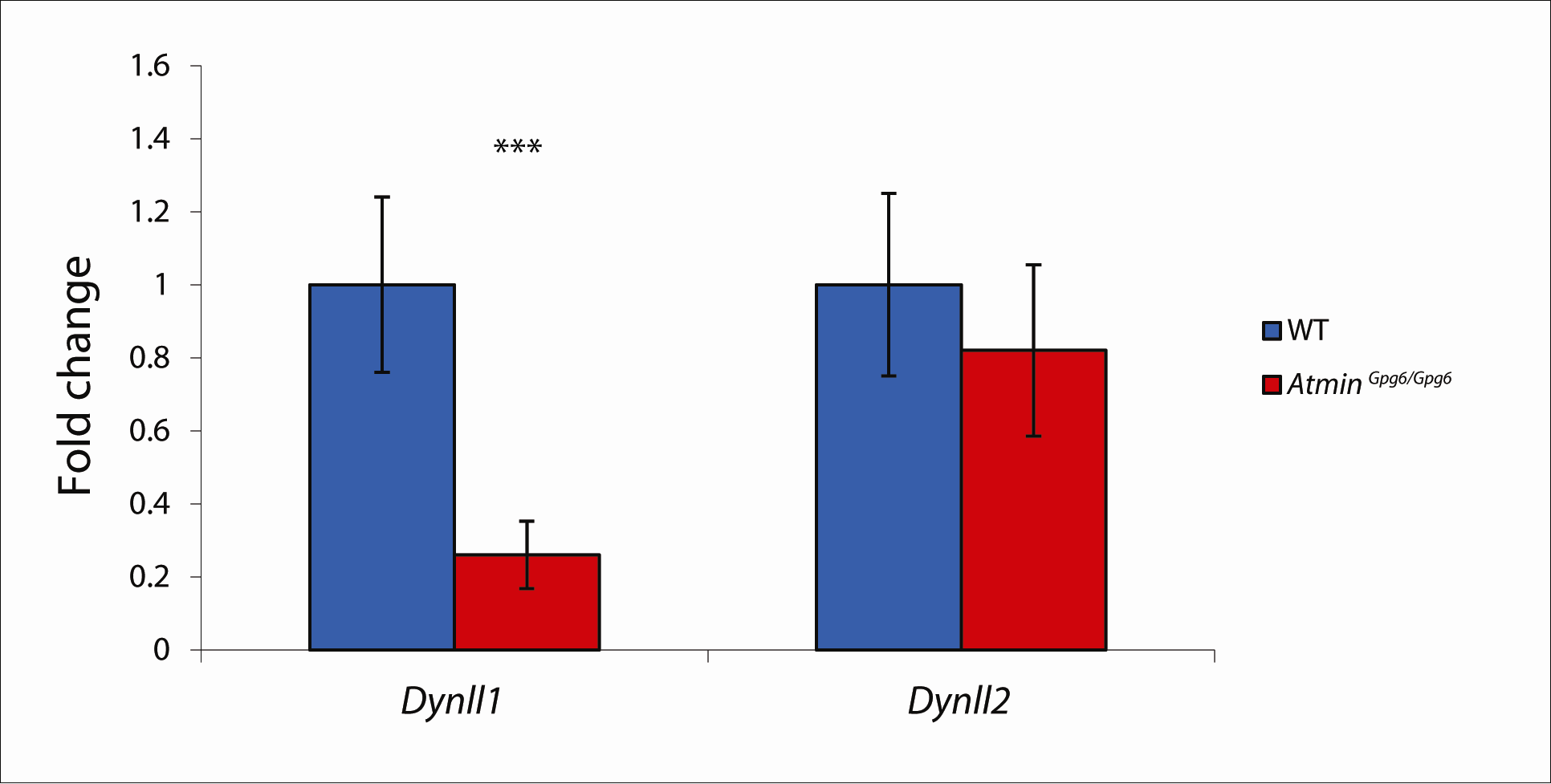


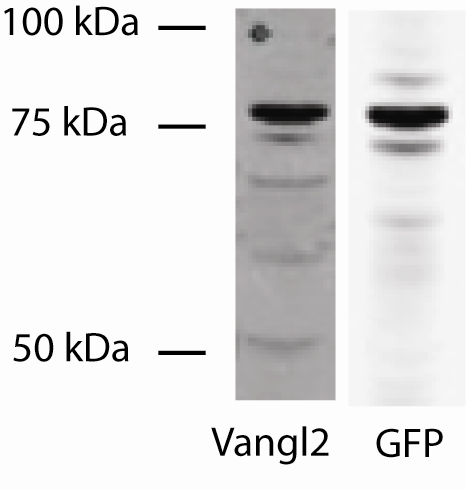


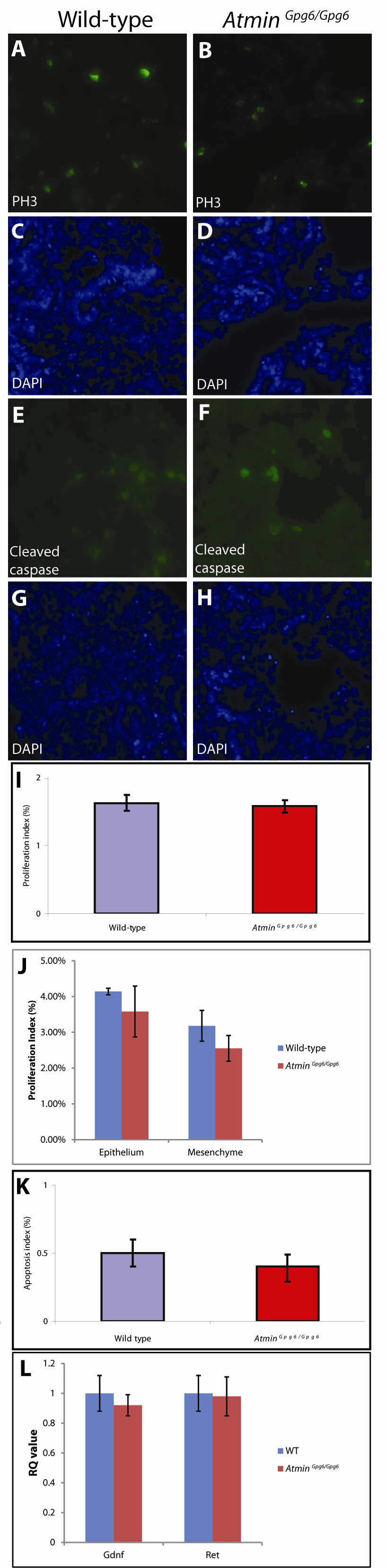

Supplement: Supplementary Data [file supp_ddu246_ddu246supp.doc]
